# A novel frequent *BRCA1* recurrent variant c.5117G > A (p.Gly1206Glu) identified after 20 years of *BRCA1/2* research in the Baltic region: cohort study and literature review

**DOI:** 10.1186/s13053-021-00168-z

**Published:** 2021-01-19

**Authors:** P. Loza, A. Irmejs, Z. Daneberga, E. Miklasevics, E. Berga-Svitina, S. Subatniece, J. Maksimenko, G. Trofimovics, E. Tauvena, S. Ukleikins, J. Gardovskis

**Affiliations:** 1 Riga Stradins University, Institute of Oncology, Riga, Latvia; 2 Pauls Stradins Clinical University Hospital, Riga, Latvia; 3 Department of Surgery, Riga Stradins University, Riga, Latvia

**Keywords:** BRCA1, BRCA2, C.5266dupC, C.4035delA, C.181 T > G, C.5117G > A (p.Gly1706Glu), C.4675G > A (p.Glu1559Lys), C.1961delA (p.Lys654fs), C.5503C > T (p.Arg1835*)

## Abstract

**Background:**

Several recent studies in the Baltic region have found extended spectrum of pathogenic variants (PV) of the *BRCA1/2* genes. The aim of current study is to analyze the spectrum of the *BRCA1/2* PV in population of Latvia and to compare common PV between populations of the Baltic region.

**Methods:**

We present a cohort of 9543 unrelated individuals including ones with cancer and unaffected individuals from population of Latvia, who were tested for three most common *BRCA1* founder PV. In second line testing, 164 founder negative high-risk individuals were tested for PV of the *BRCA1/2* using next generation sequencing (NGS). Local spectrum of the *BRCA1/2* PV was compared with the Baltic region by performing a literature review.

**Results:**

Founder PV c.5266dupC, c.4035delA or c.181 T > G was detected in 369/9543 (3.9%) cases. Other *BRCA1/2* PV were found in 44/164 (26.8%) of NGS cases. Four recurrent *BRCA1* variants c.5117G > A (p.Gly1706Glu), c.4675G > A (p.Glu1559Lys), c.5503C > T (p.Arg1835*) and c.1961delA (p.Lys654fs) were detected in 18/44 (41.0%), 5/44 (11.4%), 2/44 (4.5%) and 2/44 (4.5%) cases respectively. Additionally, 11 *BRCA1* PV and six *BRCA2* PV were each found in single family.

**Conclusions:**

By combining three studies by our group of the same cohort in Latvia, frequency of the *BRCA1/2* PV for unselected breast and ovarian cancer cases is 241/5060 (4.8%) and 162/1067 (15.2%) respectively. The frequency of three “historical” founder PV is up to 87.0% (369/424). Other non-founder PV contribute to at least 13.0% (55/424) and this proportion probably will rise by increasing numbers of the *BRCA1/2* sequencing. In relative numbers, c.5117G > A is currently the third most frequent PV of the *BRCA1* in population of Latvia, overcoming previously known third most common founder variant c.181 T > G. In addition to three *BRCA1* founder PV, a total of five recurrent *BRCA1* and two recurrent *BRCA2* PV have been reported in population of Latvia so far. Many of the *BRCA1/2* PV reported in Latvia are shared among other populations of the Baltic region.

## Introduction

Pathogenic variants of the *BRCA1* and *BRCA2* genes (*BRCA1/2*) are established as most significant genetic risk factors for developing breast and ovarian cancers. Detection of germline pathogenic variants (PV) in asymptomatic carriers allows appropriate preventive actions including imaging and risk reductive surgical procedures to be taken, which can improve early detection and prevent the development of the disease. Moreover, in individuals with breast and/or ovarian cancer, the *BRCA1/2* status serves as an important guide for surgical treatment planning as well as medical management (chemotherapy and targeted therapy with poly (ADP-ribose) polymerase inhibitors (PARPi). The impact of discovered PV reaches far beyond individual level, often evoking cascade of genetic counselling and testing of many other family members. However, the initial high cost of sequencing of whole *BRCA1/2* genes in some countries led to the concept of targeted founder PV testing that could detect majority of cases for a small fraction of cost.

The Polish publication about three *BRCA1* founder variants (c.5266dupC, c.4035delA, c.181 T > G**)** in year 2000 was supported by reports from other Baltic region countries which confirmed similar mutational spectrum [1]. The early studies between years 1999–2005 by several groups in Latvia showed that two variants, c.5266dupC and c.4035delA constitute more than 80% of all PV identified by analysis of the entire *BRCA1* gene and they contribute to 3,7–5.7% and 9.9–17.3% of all consecutive breast and ovarian cancers respectively [2–6]. The third PV, c.181 T > G, was also confirmed in the population of Latvia, however, found much less frequently than in Poland, providing a concept for inexpensive founder PV testing in the country for all consecutive breast and ovarian cancers [7]. The concept was further supported by finding that 57.5% of *BRCA1* positive cases actually did not meet family cancer history criteria to qualify for *BRCA1/2* testing [7]. Similar results of all three common founder variants have been reported also from Lithuania and Belarus [8, 9]. However, in Estonia and west Russia only two founder PV (c.5266dupC and c.4035delA*)* have been reported [10, 11]*.* For more than a decade there was scarce published data about frequency of other PV of the *BRCA1/2* in the Baltic region, which partially was related to relatively high cost of complete *BRCA1/2* testing. Taking into account high frequency of two founder PV, someone could even speculate that the number of non-founder variants is negligible. This assumption was in concordance with several other studies mentioned below.

In our previous study in year 2013, non-founder PV of the *BRCA1/2* were identified in 4 out of 30 (13%) founder-negative, high-risk breast/ovarian cancer families [7]. Around the same time, by screening 1068 breast cancer and 231ovarian cancer cases and performing complete *BRCA1/2* sequencing for a selected cohort of 160 cases, another group in Latvia concluded that the prevalence of non-founder *BRCA1/2* PV did not likely exceed 0.5% (CI 95% 0.1–0.9%) among unselected breast cancer cases and 1% (CI 95% 0–2.3%) among unselected ovarian cancer cases [5].

Summary of *BRCA1/2* variant spectrum from Latvia, Lithuania, Poland and Russia was published in year 2018 in the worldwide review by *Consortium of Investigators of Modifiers of BRCA1/2 (CIMBA*), showing common founder variants in the Baltic region, however, containing relatively small number of reported non-founder variants [12]. The increasing availability of NGS technology lead to the most recent pilot study reported by our group, which found relatively high frequency of non-founder *BRCA1/2* PV, in seven out of sixteen (44%) founder negative, high-risk breast/ovarian cancer families, including a novel recurrent pathogenic *BRCA1* variant c.5117G > A, which was found in two families in Latvia [13]. Findings of this pilot study mandated further research to reevaluate the true frequency and spectrum of non-founder PV of the *BRCA1/2*.

## Aim of the study

To study the *BRCA1/2* non-founder PV spectrum and frequency in Latvia and to compare it with published data from populations of the Baltic region.

## Materials and methods

The retrospective data analysis of 9543 patients from the population of Latvia was performed. Individuals were tested for three *BRCA1* founder PV (c.181 T > G; c.4035delA; c.5266dupC*)* from year 2004 to 2020*.* In this cohort, 4927/9543 (51.6%) had breast cancer, 1049/9543 (11.0%) had ovarian cancer, 950/9543 (10.0%) had other cancer and 2617/9543 (27.4%) had no diagnosis of cancer at the time of testing.

Any one of following criteria were applied to select patients for the *BRCA1* founder testing:

- Women with breast cancer and/or ovarian cancer regardless of age and family history
- Unaffected subjects who have first-degree or second-degree relatives with breast/ovarian cancers

In founder negative cases, any of following criteria was used to select high-risk individuals for further complete *BRCA1/2* testing by NGS or Sanger sequencing:

- Manchester scoring system of 15 or more points [14]
- Fulfil National Comprehensive Cancer network (NCCN) Hereditary Cancer Testing Criteria (*version 1.2020,**www.nccn.org*)

Using above criteria and taking into account the availability of DNA samples, 164 individuals were selected for complete *BRCA1/2* testing by NGS. This testing was carried out either by means of a commercial service or as part of a research program at the Riga Stradins University, Institute of Oncology. This group consisted of 133/164 (81.1%) cases of breast cancer, 18/164 (11.0%) cases of ovarian cancer, 3/164 (1.8%) cases of breast and ovarian cancers and 10/164 (6.1%) unaffected high-risk individuals.

Commercial NGS for the clinical purpose was available since 2016. The patient samples were analyzed with different commercially available targeted panels for hereditary cancer in different commercial medical laboratories. For this research, we analyzed only the *BRCA1* and *BRCA2* gene sequencing results, obtained from commercial testing. Apart from commercial testing, 94/171 samples were analyzed in the RSU Institute of Oncology using AmpliSeq™ BRCA Panel for Illumina® (Illumina, USA) by iSeq100 system. The minimal coverage was >100x.

All individuals provided written informed consent before genetic testing.

For literature review we searched *Pubmed* database to map the spectrum of published PV in countries of the Baltic region. We searched by entering “*BRCA1 country,*” and “*BRCA2 country*” and manually filtered out irrelevant studies. Only relevant studies containing data about pathogenic *BRCA1/2* variants from each country were selected. A total of 7/36, 7/39, 1/3, 22/386, 17/120 and 4/24 papers were selected from Latvia, Lithuania, Estonia, Poland, Russia and Belarus respectively. Recurrent PV were reviewed and summarized in the context of CIMBA worldwide review consisting of 29,700 families (Table 1) [12].

**Table 1:** Published number of families with recurrent *BRCA1/2* pathogenic variants in different Baltic region countries and worldwide

HGVS [15]	Latvia	Lithuania	Estonia	Russia	Poland	Belorussia	Worldwide (CIMBA [12])
***BRCA1*** NM_007294.3							
c.181 T > G	15 (current study) 7 [5]	22 [12] 15 [8]	–	2 [16]	276 [12]	20 [17]	Worldwide total 909
c.1961delA	2 (current study) 1 [13]	–	–	1 [18] 1 [12]	–	–	Worldwide total 79 UK10 Germany 6
c.2481delA	1 (current study)	1 [12]	–	–	–	–	–
c.3700_3704delGTAAA	1 (current study)	2 [12]	–	–	10 [19]	–	Worldwide total 159 Germany 57 Czech 25
c.4035delA	147 (current study) 40 [12] 43 [2]	113 [12] 96 [8]	3 [12] 6 [20]	11 [12]	69 [12]	17 [17]	Worldwide total 304 Germany 25
c.4675G > A	5 (current study) 1 [5], 1 [7]	1 [12]	1 [20]	–	1 [21]	–	Worldwide total 5 Germany 1 USA 1
c.4689C > G	1 (current study)	2 [12]	–	1 [12]	4 [22]	–	worldwide total 104 Germany 66
c.5095C > T	1(current study)	–	–	–	1 [22]	–	Worldwide total 37 Spain 3 Germany 13
c.5117G > A	18 (current study) 2 [13]	1 [12]	1 [20]	1 [18]	–	–	Worldwide total 18 Spain 13 UK 1
c.5251C > T	–	–	–	–	6 [19]	–	Worldwide total 94 Germany 9 Austria 16
c.5266dupC	211 (current study) 58 [5]	58 [12] 56 [8]	7 [10]	135 [12]	711 [12]	49 [17]	Worldwide total 2942
c.5346G > A	–	–	–	–	5 [19]	–	Worldwide total 3 Germany 2
c.5503C > T	2 (current study)	1 [12]	–	1 [12]	–	–	Worldwide total 183 Germany 53
c.68_69del	2 ([5]) 1 [12]	3 [23] 3 [12]	–	7 [12] 2 [24]	9 [19] 2 [12]	–	Worldwide total 2301 Germany 47
c.843_846delCTCA	1 (current study) 1 [5]	2 [12] 1 [23]	–	–	–	–	Worldwide total 54 Germany 13 Italy 10
c.-232-?_134 +?del exon 1-3del	1 (current study)	3 [8] 2 [23]	–	–	–	–	Worldwide total 11 Germany 3 USA 4
***BRCA2*** NM_000059.3							
c.3847_3848del	–	4 [12]	–	–	1 [12]	–	Worldwide total 169 Germany 36
c.3974_3975insTGCT	–	–	–	–	3 [19]	–	–
c.5946delT	1 (current study)	–	–	1 ([12])	3 [12]	–	Worldwide total 1401 USA 723 Germany 29
c.6267delG	–	–	–	–	3 [19]	–	–
c.646delG	4 [7]	–	–	–	–	–	–
c.658delGT	3 [7]	–	–	–	–	–	–
c.658_659del	–	13 [12]	–	–	1 [12] 3 [19]	–	Worldwide total 130 Germany 22
c.7913_7917delTTCCT	–	–	–	–	3 [19]	–	Worldwide total 37 Germany10 Denmark 7
c.8572C > T	1(current study)	2 [8]	–	–	–	–	Worldwide total 3 Italy 2
c.9402delC	–	–	–	–	5 [19]	–	–
c.9246_9247insA	–	–	–	–	4 [19]	–	–

## Results

Among 9543 unrelated individuals who underwent *BRCA1* founder testing, a PV was found in 369/9543 (3.9%) cases. Two variants, c.5266dupC and c.4035delA were detected in 211/369(57.2%) and 143/369 (38.8%) cases respectively. However, c.181 T > G was detected only in 15/369 (4.0%) of cases. At the time of testing, in cohort of founder positive cases, 165/369 (44.7%) had only breast cancer, 111/369 (30.0%) had only ovarian cancer, 33/369 (8.9%) had breast and ovarian cancers, 50/369 (13.5%) were unaffected individuals and 10/369 (2.7%) were affected by other cancer (colorectal, prostate, urinary tract). Founder PV rate for unselected breast and ovarian cancer cases was 198/4927 (4%) and 144/1049 (13.7%) respectively.

A total of 164/9174 (1.8%) of founder negative high-risk individuals were selected and tested by NGS. A PV of the *BRCA1/2* was detected in 44/164 (26.8%) of families, of which 38/44 (86.4%) were *BRCA1* and 6/44 (13.6%) were *BRCA2* positive. Four recurrent PV of the *BRCA1*, c.5117G > A, c.4675G > A, c.1961delA and c.5503C > T were detected in 18/44 (40.1%), 5/44 (11.4%), 2/44 (4.5%) and 2/44(4.5%) of families respectively. All families with PV c.5117G > A were of Latvian ethnicity with no established relation. Additionally, 11 different PV of the *BRCA1*, c.1961dupA, c.2241delC, c.2481delA, c.3700_3704delGTAA, c.4065delTCAA, c.4689C > G, c.5095C > T, c.5256_5278-2757del, c.843_846delCTCA, rsa17q21.31(BRCA1 5’UTR-3’UTR)× 1 (exon 1-23del) and c.-232-?_134 +?del were each found in a single family.

There were no recurrent PV of the *BRCA2* detected in this study. Six PV of the *BRCA2*, c.1310_1313delAAGA*,* c.1813dupA, c.5946delT, c.8572C > T, c.9381G > A and c.9097delA were each found in single family (Table 2).

**Table 2:** *BRCA1/2* non-founder pathogenic variants

HGVS [15]	Effect on protein	Mutation type	Mutation effect	No of Families
**Total**				**44**
***BRCA1*** NM_007294.3				**38**
c.1961delA	p.Lys654fs	FS	PTC	2
c.1961dupA ^a,b^	p.Tyr655fs	FS	PTC	1
c.2241delC ^a,b^	p.Asp749fs	FS	PTC	1
c.2481delA ^a^	Gly828fs	FS	PTC	1
c.3700_3704delGTAAA ^a^	p.Val1234fs	FS	PTC	1
c.4065_4068delTCAA ^a,b^	p.Asn1355fs	FS	PTC	1
**c.4675G > A**	p.Glu1559Lys	MS	MS	5
c.4689C > G ^a^	p.Tyr1563*	NS	PTC	1
c.5095C > T ^a^	p.Arg1699Trp	MS	MS	1
**c.5117G > A**	p.Gly1706Glu	MS	MS	18
c.5256_5278-2757del ^a,b^	–	LGR	PTC	1
c.5503C > T ^a^	p.Arg1835*	NS	PTC	2
c.843_846delCTCA	p.Ser282fs	FS	PTC	1
rsa17q21.31(BRCA1 5’UTR-3’UTR)×1, exon 1-23del ^a,b^	–	LGR	–	1
c.-232-?_134 +?del exon 1-3del ^a^	–	LGR	–	1
***BRCA2*** NM_000059.3				6
c.1310_1313delAAGA ^a,b^	p.Lys437fs	FS	PTC	1
c.1813dupA. ^a,b^	p.Ile605Asnfs	FS	PTC	1
c.5946delT ^a^	p.Ser1982fs	FS	PTC	1
c.8572C > T ^a^	p.Gln2858*	NS	PTC	1
c.9097delA ^a,b^	p.Thr3033Leufs	FS	PTC	1
c.9381G > A ^a,b^	p.Trp3127*	NS	PTC	1

*MS* missense, *NS* nonsense, *FS* frameshift, *LGR* Large gene rearrangement, *PTC* premature truncating codon

^a^reported in Latvia for the first time ^b^ reported in the Baltic region for the first time

## Discussion

Our data supports previously published data and confirms that c.5266dupC is the most common PV of the *BRCA1* in population of Latvia, followed by second most common variant, c.4035delA [3, 5, 6]. A PV c.181 T > G was found only in minority, 15/369 (4%) of all founder positive families, which is less than expected, because in previous publication by group from Latvia it was found in 6.4% of all founder positive cases [5].

Our study has the largest to date published cohort of non-founder *BRCA1/2* PV families in Latvia. By sequencing 164 probands, we found a frequent recurrent *BRCA1* missense PV c.5117G > A in 18/164 (11.0%) of *BRCA1/2* tested families. This is a significant finding, indicating that this PV is very common in population of Latvia, moreover, all carriers of this PV being Latvians. We already have reported this PV in two families in our previous smaller cohort study, which sums up to 20 families in Latvia [13]. Probable explanation of late finding of this frequent PV is that this variant has been classified as pathogenic only since year 2015, after being reviewed by the *ENIGMA BRCA1/2 expert panel* and submitted evidence to “*ClinVar*” database [25]. The loss of the *BRCA1* function caused by this PV has also been confirmed by the “*Database of Functional Classification of BRCA1 variants based on Saturation Genome Editing*” [26]. However, this PV has initially been described in year 2003 (as BIC 5236G > A) in three Spanish families by the group in Spain [27]. The author estimated that this variant, together with the other three locally recurrent variants (187^_^188delAG, 330A > G, 5242C > A, and 589^_^590del) accounted for 46.6% of all *BRCA1* detected PV in Spain. This data was further supported by large worldwide *BRCA1/2* report of 29,700 families by *CIMBA* in year 2018, where this PV was reported in 13/189 (6.9%) of Spanish *BRCA1* positive families. However, in the same report, this particular variant was generally rare globally, reported only in 2/4317 (0.05%) of *BRCA1* positive US families, and in a single family from UK, France and Lithuania [12]. This PV has also been reported in a single family in Russia and Estonia [18, 20]. More research with larger cohort is needed to assess the true frequency of this PV within the group of unselected breast/ovarian cancers. According to data from literature, Latvia and Spain have by far the largest number of families with this PV and haplotype studies between the populations should be considered to explain this unique finding.

The second significant finding from our study is a recurrent pathogenic missense variant c.4675G > A (p.Glu1559Lys) that we detected in 5/164 (3.0%) *BRCA1/2* tested families. This PV has previously been reported in two other families in Latvia, one family in Lithuania and Estonia [5, 7, 8, 20]. In a recent report by *CIMBA*, this variant was reported in only five families Worldwide, one in each country - Germany, UK, USA, Latvia and Lithuania [12]. Moreover, in another study this PV was reported in three families in Germany and one family in Poland [21, 28]. Currently Latvia has by far the highest reported absolute and relative numbers (seven unrelated families) of this PV in the world. However, there is little published data available about this PV, and we propose that more research is needed to specify its frequency in the population of the Baltic region and the world more precisely.

Another recurrent *BRCA1* pathogenic nonsense variant c.5503C > T was found in two families. It has previously been reported in 53 German, 20 British, one Lithuanian and one Russian family by *CIMBA* [12]. In publication by the Russian group, this PV was found in one subject after sequencing 95 high-risk founder negative breast cancer patients [18].

A pathogenic *BRCA1* frameshift variant c.1961delA was detected in two families. This PV was already reported in our previous study in one Latvian family [13], so currently we propose this variant as recurrent in Latvia. Similarly, previously reported PV of the *BRCA1* c.843_846delCTCA has been found in another family from our current study, also rendering this PV locally recurrent [5].

Eleven PV of the *BRCA1* (c.1961dupA, c.2241delC, c.2481delA, c.3700_3704delGTAAA, c.4065_4068delTCAA, c.4689C > G, c.5095C > T, c.5503C > T, c.5256_5278-2757del, c.-232-?134 +?del, rsa17q21.31 (BRCA1 5’UTR-3’UTR)× 1 deletion encompassing whole *BRCA1* gene) and all 6 PV of the *BRCA2* from our current study are reported first time in Latvia (Table 2). However, eight of these variants have previously been reported in the Baltic region of which five have been reported in Lithuania (Table 1). Nine PV, including five of the BRCA1 and four of the BRCA2 have been reported in the Baltic region for the first time (Table 2).

For the *BRCA1/2* we have found 13 PV common between Latvia and Lithuania, 11 PV common between Latvia and Russia, 8 PV common between Latvia and Poland and 8 PV common between Lithuania and Poland. We only found 4 PV common between Latvia and Estonia, however, there is scarce published data available as we could find only one Estonian study and some data from *dbSNP* database [10, 20]. This underscores the close genetic, ethnic, historical and geographical relationships among the populations of the Baltic region. According to genetic constitution analysis of 3012 individuals from Europe by the group from Estonia, the genetic structures of populations of Latvia, Estonia and Lithuania have major similarities and a significant overlap exists with populations of Poland and western Russia, forming a distinct Baltic region cluster [29]. It is also not surprising that several recurrent variants are shared with Germany, including three it’s most frequent ones (c.5266dupC, c.181 T > G, c.4689C > G) as it has very long historical and geographical relationships with Baltic region too. At the same time someone could expect more common variants with another Baltic region country- Sweden, as only one Slavic founder mutation c.5266dupC is shared among the most frequent ones [12]. Over the last century, the ethnic structure of population of Latvia has changed significantly. Before the Second World War, in 1935, the proportions of Latvians, Russians, Jews, Germans, Poles, Belarussians, Lithuanians, Estonians were 76.9, 8.8, 4.9, 3.3, 2.6, 1.4 1.2, 0.4% respectively, and this proportion changed to 52.0, 34.0, 0.9, 0.1, 2.3, 4.5, 1.3, 0.1% in 1989 and subsequently to 62.0, 25.4, 0.2, 0.1, 2.1, 3.3, 1.2, 0.1% in 2017 [30]. As a result, a lot of current young generation Latvians have at least some genetic imprint from Russians, Jews, Germans and Poles.

One of the drawbacks of this study is still a relatively small number (*n* = 164) of founder negative high-risk families that we have sequenced.

## Conclusions

In summary, by combining all three studies of the *BRCA1/2* reported by our group, which are covering the same cohort, the PV frequency for unselected breast and ovarian cancer cases is 241/5060 (4.8%) and 162/1067 (15.2%) respectively. The prevalence of three “historical” founder PV in population of Latvia is up to 87.0% (369/424) of all PV of the *BRCA1/2*. Other PV contribute to at least 13.0% (55/424) of total *BRCA1/2* PV, but the true spectrum and frequency of *BRCA1/2* PV still have to be defined more accurately by increasing the availability of complete *BRCA1/2* testing. However, already now we are able to confirm that at least one in 10 high-risk cases would be underdiagnosed if complete *BRCA1/2* testing is not routinely available.

We have identified five locally novel recurrent PV of the *BRCA1* of which c.5117G > A and c.4675G > A are unexpectedly frequent. Findings of our study have very practical implications as addition of those 2 recurrent variants to the *BRCA1/2* screening test potentially will cover up to 94% of all presently known PV of the *BRCA1/2* in Latvia. These 2 PV together with other recurrent PV of the *BRCA1*, three from current study (c.5503C > T, c.1961delA c.843_846delCTCA) and one previously reported recurrent PV, c.68_69del, along with two recurrent PV of the *BRCA2* from our previous study (c.646delG and c.658delGT) should be considered for inclusion in the local *BRCA1/2* founder screening kit. Additionally, 5 PV of the *BRCA1 (*c.2481delA, c.3700_3704delGTAAA, c.4689C > G, c.5095C > T, c.-232-?_134 +?del) and 2 PV of the *BRCA2 (*c.5946delT, c.8572C > T) that we found each in a single family in Latvia, have been reported in neighboring countries and hence could also be candidates for inclusion in local *BRCA1/2* screening kit.

Many common *BRCA1/2* pathogenic variants are shared between populations of the Baltic region, however still miniscule absolute numbers of tested families prevent the formation of complete picture. Interestingly, PV c.5117G > A and c.4675G > A have not been reported as recurrent in neighboring countries and there are only isolated cases reported from other populations of the Baltic region. Accordingly this finding underscores the possibility of finding new relatively frequent recurrent PV in populations, including neighboring ones, where limited number of cases have been tested for complete *BRCA1/2* so far.

According to the data of our study some corrections about five most frequent *BRCA1* mutations in Latvia should be done to complement the earlier published data by CIMBA: Most frequent-c.5266dupC, second -c.4035delA, third- 5117G > A, fourth- c.181 T > G, fifth- c.4675G > A.

## Data Availability

The datasets generated and/or analyzed during the current study are available from the corresponding author on request.
